# Long noncoding RNA *ANCR* inhibits the differentiation of mesenchymal stem cells toward definitive endoderm by facilitating the association of PTBP1 with *ID2*

**DOI:** 10.1038/s41419-019-1738-3

**Published:** 2019-06-24

**Authors:** Jing Li, Yanlei Yang, Junfen Fan, Haoying Xu, Linyuan Fan, Hongling Li, Robert Chunhua Zhao

**Affiliations:** Institute of Basic Medical Sciences Chinese Academy of Medical Sciences, School of Basic Medicine Peking Union Medical College, Peking Union Medical College Hospital, Beijing Key Laboratory of New Drug Development and Clinical Trial of Stem Cell Therapy (BZ0381), 100005 Beijing, China

**Keywords:** Adult stem cells, Mesenchymal stem cells

## Abstract

The generation of definitive endoderm (DE) cells in sufficient numbers is a prerequisite for cell-replacement therapy for liver and pancreatic diseases. Previously, we reported that human adipose-derived mesenchymal stem cells (hAMSCs) can be induced to DE lineages and subsequent functional cells. Clarifying the regulatory mechanisms underlying the fate conversion from hAMSCs to DE is helpful for developing new strategies to improve the differentiation efficiency from hAMSCs to DE organs. Long noncoding RNAs (lncRNAs) have been shown to play pivotal roles in developmental processes, including cell fate determination and differentiation. In this study, we profiled the expression changes of lncRNAs and found that antidifferentiation noncoding RNA (*ANCR*) was downregulated during the differentiation of both hAMSCs and embryonic stem cells (ESCs) to DE cells. *ANCR* knockdown resulted in the elevated expression of DE markers in hAMSCs, but not in ESCs. *ANCR* overexpression reduced the efficiency of hAMSCs to differentiate into DE cells. Inhibitor of DNA binding 2 (*ID2*) was notably downregulated after *ANCR* knockdown. *ID2* knockdown enhanced DE differentiation, whereas overexpression of *ID2* impaired this process in hAMSCs. *ANCR* interacts with RNA-binding polypyrimidine tract-binding protein 1 (PTBP1) to facilitate its association with *ID2* mRNA, leading to increased *ID2* mRNA stability. Thus, the *ANCR*/PTBP1/*ID2* network restricts the differentiation of hAMSCs toward DE. Our work highlights the inherent discrepancies between hAMSCs and ESCs. Defining hAMSC-specific signaling pathways might be important for designing optimal differentiation protocols for directing hAMSCs toward DE.

## Introduction

Definitive endoderm (DE) is derived the from mesendoderm^1,2^ and is a crucial stage in early embryogenesis and can commit to organs including the thyroid, lungs, pancreas, liver, and intestines^3^. The generation of DE and DE-derived lineages from a variety of genetic backgrounds would be beneficial not only for regenerative medicine applications but also for drug testing and toxicology studies.

Numerous protocols have been set up to direct embryonic stem cells (ESCs) or induced pluripotent stem cells (iPSCs) to differentiate into DE, providing valuable paradigms for the design of induction strategies^4,5^. However, the ethical/legal issues, safety concerns, and risks of teratoma formation hinder their utilization as starting materials in translational medicine. Adult stem cells, such as mesenchymal stem cells (MSCs), hold great promise for clinical applications due to their easy isolation, freedom from ethical issues, hypoimmunogenicity, and multipotent differentiation capacity^6,7^. MSCs derived from adipose, bone marrow, dental pulp, and umbilical cord have been reported to successfully differentiate into DE^8–10^, hepatocyte-like^11–14^ and β-cell-like^8,15,16^ cells. However, the existing differentiation protocols for directing adult stem cells to DE have mostly been developed by directly mimicking embryonic development programs from primitive streak to DE and its derivative’s lineages. Inherent differences between ESCs/iPSCs and MSCs imply that they may undergo different processes toward DE. Thus, understanding the molecular mechanisms specific for DE differentiation from MSCs might help to create more desirable procedures to obtain DE-derived lineages in vitro.

Long noncoding RNAs (lncRNAs) are a subset of RNAs that are longer than 200 nt, have mRNA structure, and are usually polyadenylated but rarely encode proteins^17^. Increasing studies have reported that lncRNAs play multiple roles in the regulation of development and differentiation. Recent studies have confirmed that lncRNAs participate in the differentiation of all three germ layers, including in muscle^18^, lung^19^, dendritic cells^20^, cardiovascular^21^, neural^22^, and epidermal tissue^23^. LncRNAs also have been reported to play critical roles in the DE differentiation of ESCs. For example, lncRNA *DENAR1* functions in human endoderm differentiation by regulating FOXA2 expression^24^. The lncRNA *DIGIT*, a conserved lncRNA in mouse and human, regulates GSC expression to promote DE differentiation in ESCs^25^. To date, little is known about the functions of lncRNAs in the differentiation of MSCs to DE.

In this study, we profiled lncRNA expression during human adipose tissue-derived mesenchymal stem cells (hAMSCs) differentiation into DE and found a set of DE differentiation-related lncRNAs. Among them, the lncRNA *ANCR* (antidifferentiation ncRNA, or DANCR) was significantly downregulated. *ANCR* was previously found to promote progenitor maintenance and prevent differentiation in epidermal progenitors^23^, osteoblasts^26,27^, and chondrogenesis^28,29^. However, the role of *ANCR* in the fate conversion of hAMSCs toward DE remains to be discovered. Herein, we provide evidence that *ANCR* could inhibit the differentiation of hAMSCs to DE by increasing the mRNA stability of *ID2* through facilitating its binding with polypyrimidine tract-binding protein 1 (PTBP1).

## Results

### *ANCR* was dramatically downregulated during the differentiation of hAMSCs to DE

**Fig. 1:**
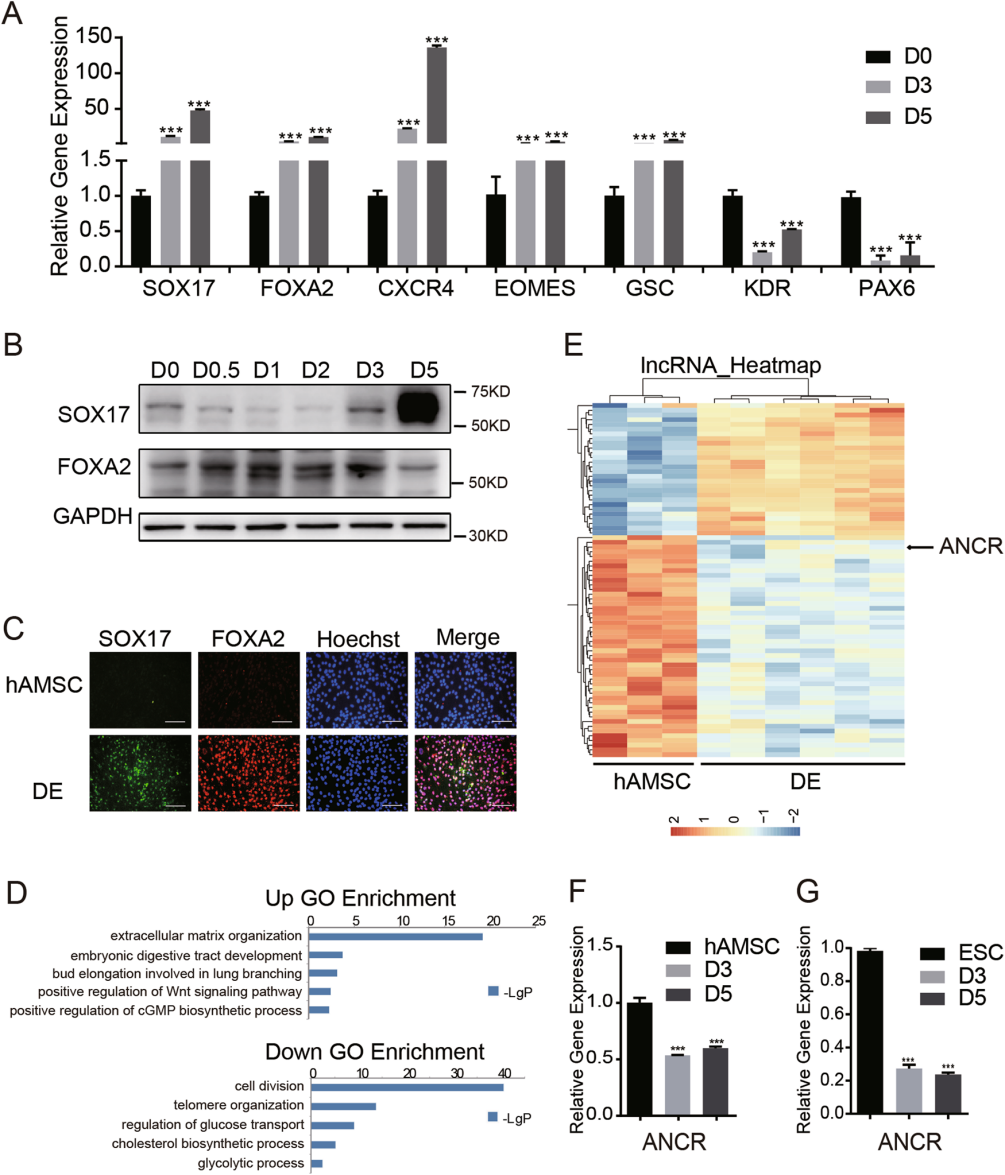
*ANCR* was dramatically downregulated during the differentiation of hAMSCs to DE. **a** qRT-PCR analysis for DE marker genes (*SOX17*, *FOXA2*, and *CXCR4*), mesendoderm marker genes (*EOMES* and *GSC*), mesoderm marker *KDR* and the ectoderm marker *PAX6* in hAMSCs on days 0, 3, and 5 after DE induction. **b** The western blot assay for DE markers (SOX17 and FOXA2) in hAMSCs at the indicated time points after DE induction. **c** Immunofluorescence (IF) staining for DE markers (SOX17 and FOXA2) in *ANCR*-knockdown hAMSCs or control hAMSCs after DE induction. The nuclei were stained with Hoechst 33342. Scale bar = 100 μm. **d** Gene Ontology (GO) analysis on the commonly upregulated (top) or downregulated (bottom) coding genes in three donors derived from hAMSCs after DE induction at day 5 compared to hAMSCs at day 0. The *y*-axis shows the GO terms and the *x*-axis shows the statistical significance (negative logarithm of *p* value). **e** Hierarchical clustering of significantly changed lncRNA on day 3 or 5 after induction compared with day 0 in matched hAMSCs from three donors. **f**, **g** qRT-PCR analysis of *ANCR* levels in hAMSC (**f**) and ESC (**g**) at the indicated time points after DE induction. Data are shown as the means ± S.D. (*n* = 3). *p < 0.05, **p < 0.01, and ***p < 0.001

We previously established a stepwise protocol using the combination of Activin A and Wnt3a to generate DE from hAMSCs^8,30^. Teo et al.^31^ reported that compared to high doses of Wnt3a, the glycogen synthase kinase-3 inhibitors Chir99021 can induce DE formation from ESCs with comparable efficiency and lower cost. Thus, we set to determine whether Chir99021 could replace Wnt3a in our protocol. As shown in Supplementary Fig. 1, the combination of 5 ng/ml Activin A and 0.3 mM Chir99021 (AC) exhibited a higher expression of key DE marker genes, including *SOX17* and *FOXA2*, compared to our previous combination of 5 ng/ml Activin A and 10 ng/ml Wnt3a (AW). Therefore, we adopted the AC protocol for the induction of DE in the subsequent study. As verified by qRT-PCR, the expression of the DE-specific genes *SOX17*, *FOXA2*, and *CXCR4*^5^ were remarkably elevated during DE differentiation (Fig. 1a). Meanwhile, the expression of mesendoderm-related genes, such as *EOMES* and *GSC*, were also upregulated (Fig. 1a), while the ectoderm marker *PAX6* as well as the mesoderm marker *KDR* were downregulated (Fig. 1a). Western blot also confirmed the upregulation of SOX17 and FOXA2 after DE induction in hAMSCs (Fig. 1b). Immunofluorescence staining (IF) revealed that double-positive FOXA2/SOX17 cells appeared after DE induction (Fig. 1c). Altogether, these data demonstrated that the AC protocol is effective in converting hAMSCs toward DE, as we reported previously^8,30^.

To identify functional lncRNAs involved in DE differentiation, we performed microarray analyses on days 0, 3, and 5 after DE induction in hAMSCs. Gene Ontology (GO) enrichment analysis of the differentially expressed coding genes indicated that genes involved in extracellular matrix organization, digestive tract development, and the Wnt signaling pathway were significantly changed (Fig. 1d). Next, we analysed the lncRNA expression profiles according to stringent criteria (fold change ≥2, expression value ≥3, and *p* value < 0.05). We identified 75 lncRNAs (28 upregulated and 47 downregulated) that were differentially expressed in DE cells versus hAMSCs (Fig. 1e). Among the top downregulated lncRNAs in hAMSCs, we noticed that the expression of the lncRNA *ANCR*, previously reported to play important role in restricting epithelium differentiation, was dramatically decreased during DE differentiation. qRT-PCR also confirmed that *ANCR* expression levels were decreased in the induced cells (Fig. 1f).

We next induced ESC differentiation toward DE cells using a well-established protocol^4^ and examined the expression of *ANCR* during this progress. The induction efficiency was confirmed by qRT-PCR and IF staining (Supplementary Fig. 2). We found that *ANCR* expression levels were continuously reduced during the differentiation of ESCs toward DE (Fig. 1g). Thus, we focused on the role of *ANCR* in the generation of DE from hAMSCs as well as ESCs.

### *ANCR* knockdown improved the differentiation of hAMSCs to DE

**Fig. 2:**
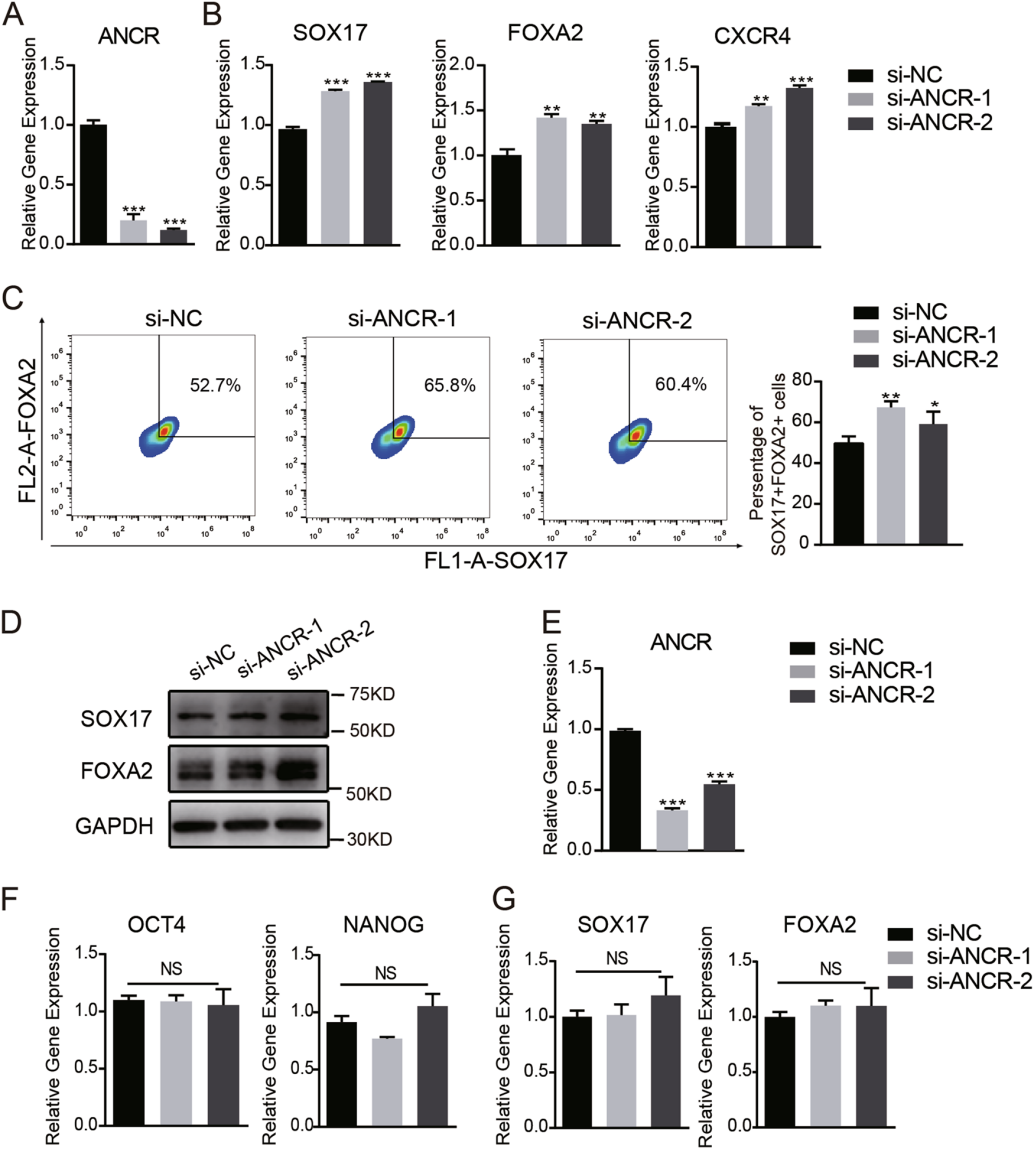
*ANCR* knockdown improved the differentiation of hAMSCs to DE. **a** *ANCR* was silenced in hAMSCs using two independent siRNAs (si-*ANCR*-1 and si-*ANCR*-2). The knockdown efficiency was verified by qRT-PCR compared with the negative control (NC). **b** qRT-PCR analysis detected DE marker genes (*SOX17*, *FOXA2*, and *CXCR4*) in *ANCR*-knockdown hAMSCs or control hAMSCs on day 5 after DE induction. **c** Flow cytometry analysis of the FOXA2^+^/SOX17^+^ subpopulation in *ANCR*-knockdown hAMSCs or control hAMSCs after DE induction. **d** Western blot detected DE markers (SOX17 and FOXA2) in *ANCR*-knockdown hAMSCs or control hAMSCs on day 5 after DE induction. **e** *ANCR* was silenced in ESCs using two independent siRNAs (si-ANCR-1 and si-ANCR-2). The knockdown efficiency was verified by qRT-PCR compared with the negative control (NC). **f**, **g** qRT-PCR analysis of detected stem cell markers genes (*OCT4* and *NANOG*) from ESCs (**f**) and DE marker genes (*SOX17* and *FOXA2*) after DE induction (**g**) in *ANCR*-knockdown ESCs or control ESCs. Data are shown as the means ± S.D. (*n* = 3). *p < 0.05, **p < 0.01, and ***p < 0.001

To evaluate the effects of *ANCR* in the generation of DE, we silenced *ANCR* in hAMSCs using two pairs of specific siRNAs (Fig. 2a). After 5 days of induction, *ANCR* knockdown resulted in higher expression of the DE markers *SOX17*, *FOXA2*, and *CXCR4* compared with control siRNA-transfected cells (Fig. 2b). Flow cytometry analysis also indicated that the FOXA2/SOX17 double-positive population were higher in *ANCR*-downregulated cells (Fig. 2c). Western blot analysis consistently validated that *ANCR* depletion enhanced the expression of the DE markers FOXA2 and SOX17 after DE induction (Fig. 2d).

To investigate whether *ANCR* plays a similar role in the differentiation of ESCs towards DE, we silenced *ANCR* in ESCs with the above siRNAs (Fig. 2e). We found that *ANCR* knockdown neither changed the expression of the stemness markers *OCT4* and *NANOG* in ESCs nor the DE markers *SOX17* and *FOXA2* after DE induction compared to control cells (Fig. 2f, g). These data demonstrated that *ANCR* hampered the differentiation of DE in hAMSCs but not in ESCs, which may be because different pathways work in ESCs and hAMSCs. Likewise, BMP and bFGF, which are required for the generation of DE from ESCs^4,32–34^, were dispensable or detrimental to the generation of DE from hAMSCs (Supplementary Fig. 3).

### Overexpression of *ANCR* inhibited the differentiation of hAMSCs to DE

**Fig. 3:**
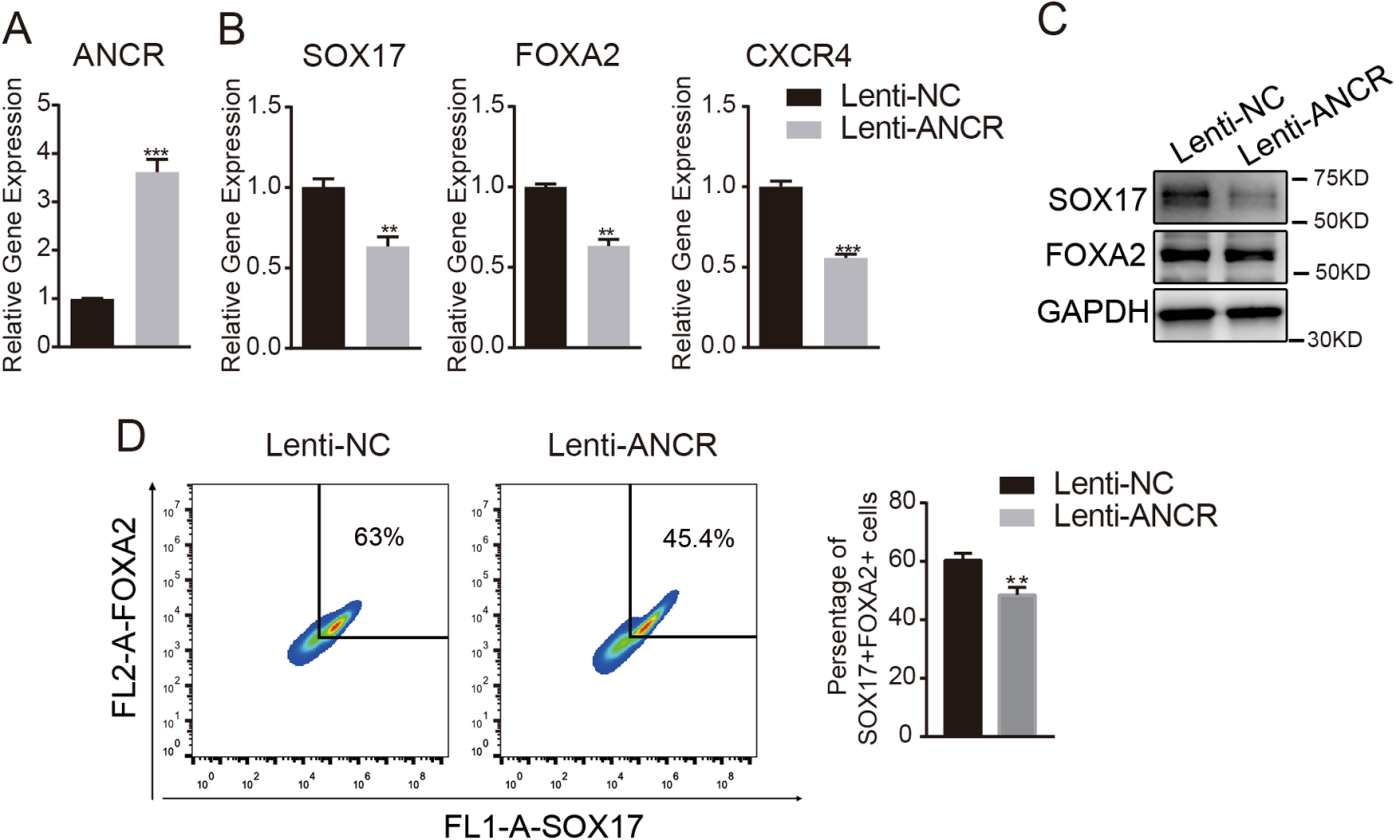
Overexpression of *ANCR* inhibited the differentiation of hAMSCs to DE. **a** hAMSCs were transduced with lentivirus overexpressing *ANCR* (Lenti-ANCR) or empty vectors (Lenti-NC). The efficiency of ectopic expression was verified by qRT-PCR. **b** qRT-PCR analysis for DE marker genes (*SOX17*, *FOXA2*, and *CXCR4*) in *ANCR*-overexpressing hAMSCs or control hAMSCs on day 5 after DE induction. **c** The western blot assay for DE markers (SOX17 and FOXA2) in *ANCR*-overexpressing hAMSCs or control hAMSCs on day 5 after DE induction. **d** Flow cytometry analysis of the FOXA2^+^/SOX17^+^ subpopulation in *ANCR*-overexpressing hAMSCs or control hAMSCs after DE induction. Data are shown as the means ± S.D. (*n* = 3). *p < 0.05, **p < 0.01, and ***p < 0.001

To further confirm the role of *ANCR* in the differentiation of hAMSCs to DE, we stably overexpressed full-length *ANCR* in hAMSCs using lentivirus (Fig. 3a). Ectopic expression of *ANCR* significantly impaired the expression of the DE markers *SOX17*, *FOXA2*, and *CXCR4*, as verified by qRT-PCR (Fig. 3b). The western blot assay revealed that overexpression of *ANCR* (Lenti-ANCR) notably delayed DE differentiation compared to empty vector control-infected cells (Lenti-NC), as indicated by decreased protein levels of the DE markers SOX17 and FOXA2 (Fig. 3c). Flow cytometry analysis also indicated that the FOXA2/SOX17 double-positive population was reduced in *ANCR*-overexpressing cells (Fig. 3d). Collectively, these data indicated that *ANCR* plays a negative role in the differentiation of hAMSCs toward DE.

### *ANCR* regulated *ID2* mRNA stability

**Fig. 4:**
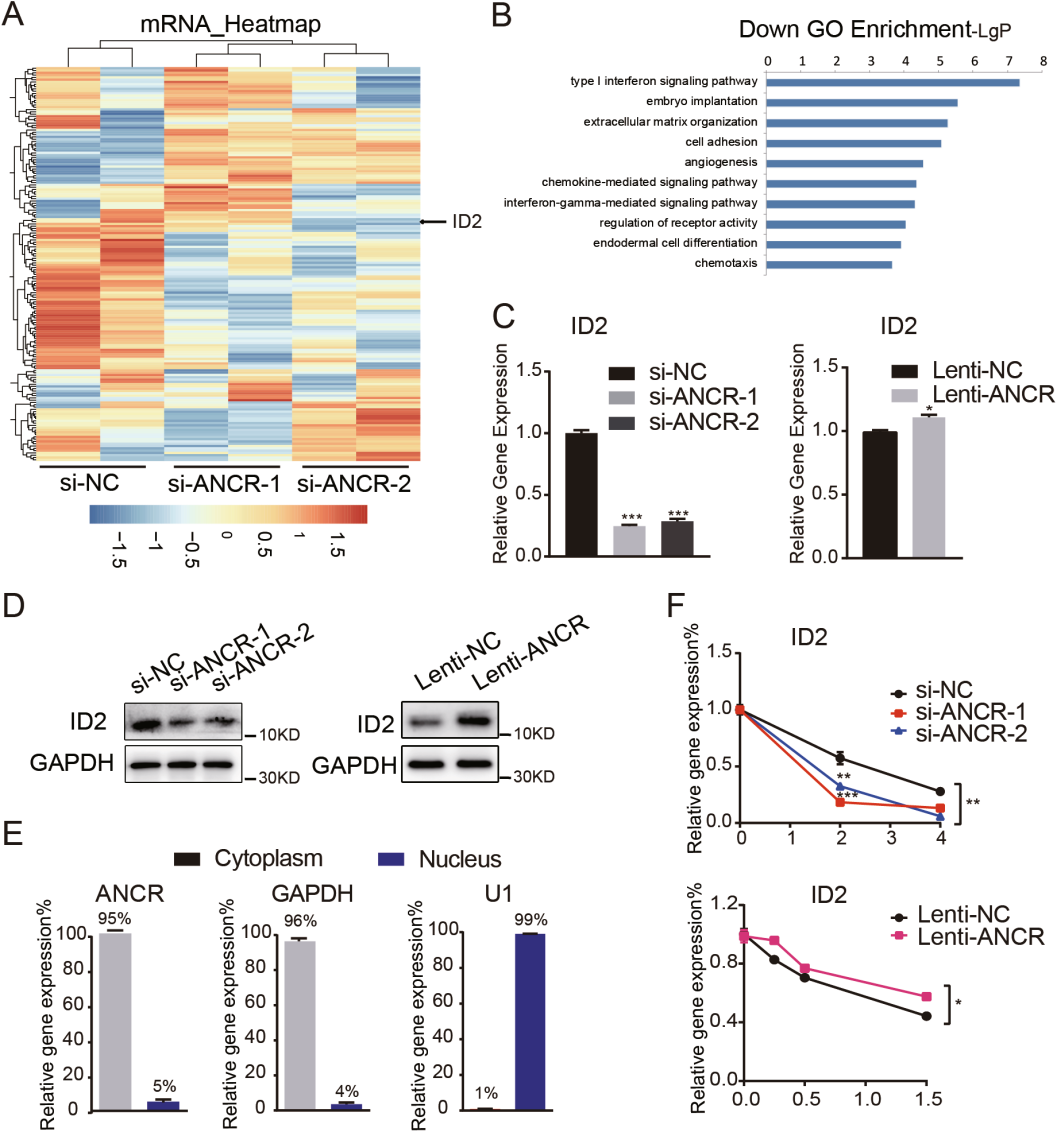
*ANCR* regulated the expression of *ID2*. **a** Microarray analysis was performed in hAMSCs transfected with si-ANCR1, si-ANCR2, and si-NC after 48 h. The heatmap represented 172 differentially expressed genes after siRNA-mediated *ANCR* knockdown (fold change ≥2, expression value≥4, and *p* value < 0.05). The black arrowhead denotes *ID2* in hAMSCs transfected with si-ANCR versus si-NC. **b** Enriched GO terms for *ANCR*-affected genes. The *y*-axis shows GO terms and the *x*-axis shows statistical significance (negative logarithm of *p* value). **c** qRT-PCR analysis of *ID2* in hAMSCs transfected with si-ANCR versus si-NC (left) or in hAMSCs overexpressing *ANCR* (Lenti-ANCR) versus empty vectors (Lenti-NC) (right). **d** Western blot detected the expression of *ID2* in hAMSCs with *ANCR* knockdown (left) and overexpression (right). **e** Fractionation of *ANCR* in hAMSCs followed by qRT-PCR. *GAPDH* served as cytoplasmic mRNA control. *U1* served as a nuclear RNA control. **f** mRNA decay detected by qRT-PCR in *ANCR-*knockdown hAMSCs (top), overexpression hAMSCs (bottom), and their corresponding controls. Data are shown as the means ± S.D. (*n* = 3). *p < 0.05, **p < 0.01, and ***p < 0.001

To investigate the mechanism by which *ANCR* impacted DE differentiation in hAMSCs, we performed microarray analysis to compare the gene expression profiles in *ANCR*-knockdown hAMSCs and control cells. The data showed that 172 genes were differentially expressed after *ANCR* knockdown (Fig. 4a) (fold change ≥2 and *p* < 0.05). GO enrichment analysis revealed that the differentially expressed genes were significantly enriched for endoderm differentiation (Fig. 4b). Among the most significantly changed genes, we focused on inhibitor of DNA binding 2 (*ID2*), which was notably downregulated after *ANCR* knockdown (Fig. 4a). The ID family has been reported to play key roles in fate determination, inhibition of differentiation, and maintenance of self-renewal in multipotent stem cells^35,36^. We then aimed to validate whether *ANCR* could regulate *ID2* levels in hAMSCs. As shown in Fig. 4c, *ANCR* silencing notably reduced the expression of *ID2* and *ANCR* overexpression slightly upregulated its expression. The western blot assay also verified the positive correlation between *ANCR* and ID2 (Fig. 4d).

Specific subcellular localization and cell fractionation are essential for understanding the function and mechanism of lncRNAs. Since *ANCR* largely displayed a cytoplasmic distribution (>90%) (Fig. 4e), we speculate that *ANCR* might promote downstream effectors at the posttranscriptional level. Thus, we next determined whether *ANCR* could regulate *ID2* mRNA decay. To evaluate the effects of *ANCR* on *ID2* mRNA stability, hAMSCs transduced with Lenti-ANCR or control vector (Lenti-NC) were treated with actinomycin D (5 μg/ml), which inhibits RNA polymerase and blocks transcription. The half-life of *ID2* mRNA was shorter than 2h based on our preliminary results (data not shown). Knockdown of *ANCR* resulted in a shortening in the half-life of *ID2* mRNA, whereas overexpression of *ANCR* prolonged the *ID2* mRNA half-life (Fig. 4f), indicating that *ANCR* stabilized *ID2* mRNA. Collectively, these data revealed that *ANCR* positively regulated the expression of *ID2* at least partly by regulating its mRNA stability.

### *ID2* negatively regulated the differentiation of hAMSCs toward DE

**Fig. 5:**
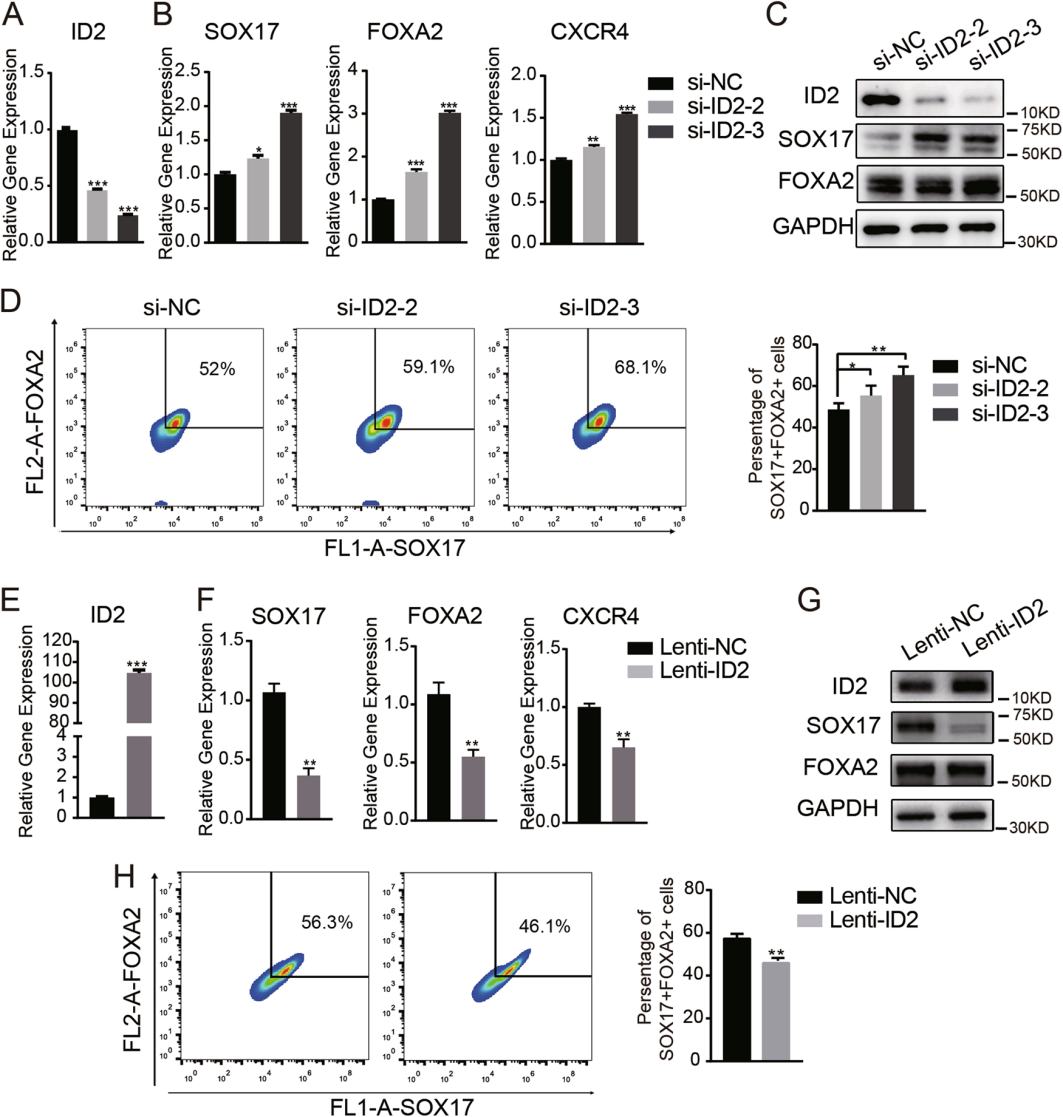
*ID2* negatively regulates the differentiation of hAMSCs to DE. **a** *ID2* was silenced in hAMSCs using two independent siRNAs (si-ID2-2 and si-ID2-3). The knockdown efficiency was verified by qRT-PCR compared with the negative control (NC). **b** qRT-PCR analysis detected DE marker genes (*SOX17*, *FOXA2*, and *CXCR4*) in *ID2*-knockdown hAMSCs and control hAMSCs on day 5 after DE induction. **c** Western blot detected the expression of ID2, SOX17, and FOXA2 in ID2-knockdown hAMSCs and control hAMSCs on day 5 after DE induction. **d** Flow cytometry analysis of the FOXA2^+^/SOX17^+^ subpopulation in si-ID2 hAMSC or si-NC hAMSCs on day 5 after DE induction. **e** hAMSCs were transduced with lentivirus overexpressing *ID2* (Lenti-ID2) or empty vectors (Lenti-NC). The efficiency of ectopic expression was verified by qRT-PCR. **f** qRT-PCR analysis detected DE marker genes (*SOX17*, *FOXA2*, and *CXCR4*) in Lenti-ID2 hAMSCs or Lenti-NC hAMSCs on day 5 after DE induction. **g** Western blot detected the expression of ID2, SOX17, and FOXA2 in Lenti-ID2 hAMSCs or Lenti-NC hAMSCs on day 5 after DE induction. **h** Flow cytometry analysis of the FOXA2^+^/SOX17^+^ subpopulation in lenti-ID2 hAMSCs or Lenti-NC hAMSCs on day 5 after DE induction. Data are shown as the means ± S.D. (*n* = 3). *p < 0.05, **p < 0.01, and ***p < 0.001

To investigate the role of *ID2* during the differentiation of hAMSCs to DE, we first silenced the expression of *ID2* using two siRNAs against *ID2* (Fig. 5a). Knockdown of *ID2* resulted in an elevated induction of the DE markers *SOX17*, *FOXA2*, and *CXCR4* after DE induction compared to control cells (Fig. 5b). Consistently, The western blot assay confirmed that *ID2* depletion was associated with increased expression of DE markers (Fig. 5c). Moreover, flow cytometry analysis also showed that the percentage of FOXA2/SOX17 double-positive cells was elevated in *ID2-*depleted cells after DE induction (Fig. 5d).

We then stably overexpressed *ID2* in hAMSCs using lentivirus transduction and verified the results by qRT-PCR (Fig. 5e). After DE induction, the expression levels of the DE markers *SOX17*, *FOXA2*, and *CXCR4* were reduced in *ID2*-overexpressing hAMSCs (Lenti-ID2) compared with control cells (Lenti-NC) (Fig. 5f). Consistently, the protein levels of SOX17 and FOXA2 were decreased in *ID2*-overexpressing cells after DE induction (Fig. 5g). Flow cytometry analysis also confirmed that DE differentiation was suppressed after *ID2* overexpression (Fig. 5h). Collectively, these data demonstrated that *ID2* negatively regulated the DE differentiation of hAMSCs, which was consistent with the function of *ANCR*.

### *ID2* was responsible for *ANCR*-mediated DE differentiation in hAMSCs

**Fig. 6:**
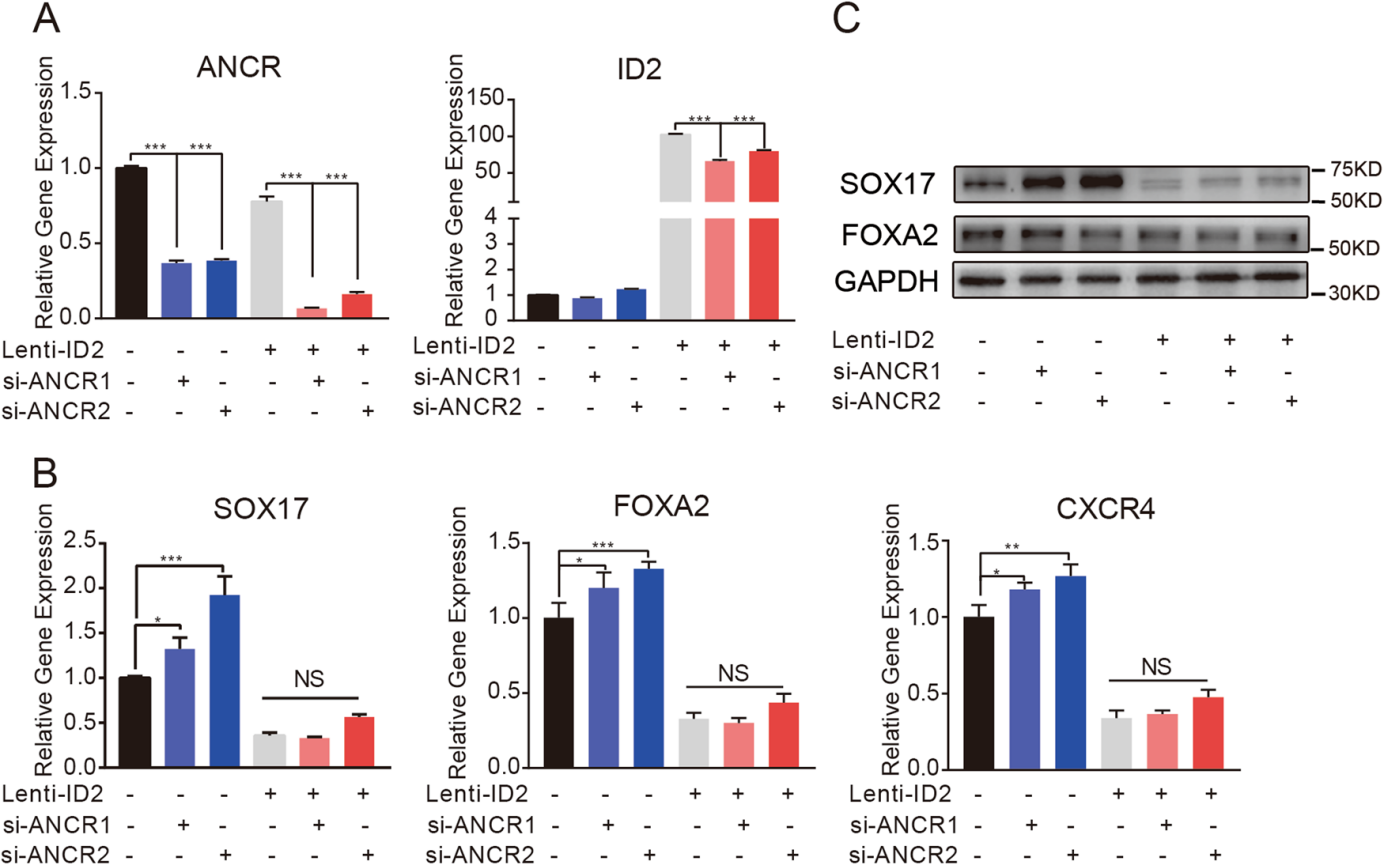
*ID2* was responsible for *ANCR*-mediated DE differentiation in hAMSCs. **a** Lenti-ID2 hAMSCs and Lenti-NC hAMSCs were transfected with siRNAs targeting ANCR or NC. The levels of *ANCR* and *ID2* were detected by qRT-PCR. **b** qRT-PCR analysis detected DE marker genes (*SOX17*, *FOXA2*, and *CXCR4*) in Lenti-ID2 hAMSCs and Lenti-NC hAMSCs transfected with siRNAs targeting ANCR or NC, respectively, on day 5 after DE induction. **c** Western blot detected the expression of SOX17 and FOXA2 in Lenti-ID2 hAMSCs and Lenti-NC hAMSCs that transfected with siRNAs targeting ANCR or NC, respectively, on day 5 after DE induction. Data are shown as the means ± S.D. (*n* = 3). *p < 0.05, **p < 0.01, and ***p < 0.001

To examine whether *ANCR* regulated DE differentiation of hAMSCs in an *ID2*-dependent manner, we overexpressed *ID2* in *ANCR*-knockdown hAMSCs or control cells (Fig. 6a). As mentioned above, *ANCR* knockdown resulted in higher expression of DE markers, whereas *ID2* overexpression could significantly decrease DE marker expression. Overexpression of *ID2* could decrease DE marker expression even under *ANCR* knockdown, indicating that *ID2* strongly inhibited DE differentiation. The promoting effects of *ANCR* knockdown on DE differentiation of hAMSCs were partially reversed when *ID2* was overexpressed, as indicated by the downregulated the expression of key DE markers (Fig. 6b, c). These data demonstrated that *ANCR* regulates hAMSCs differentiation to DE in an *ID2*-dependent manner.

### *ANCR* regulates *ID2* mRNA stability by binding to PTBP1

**Fig. 7:**
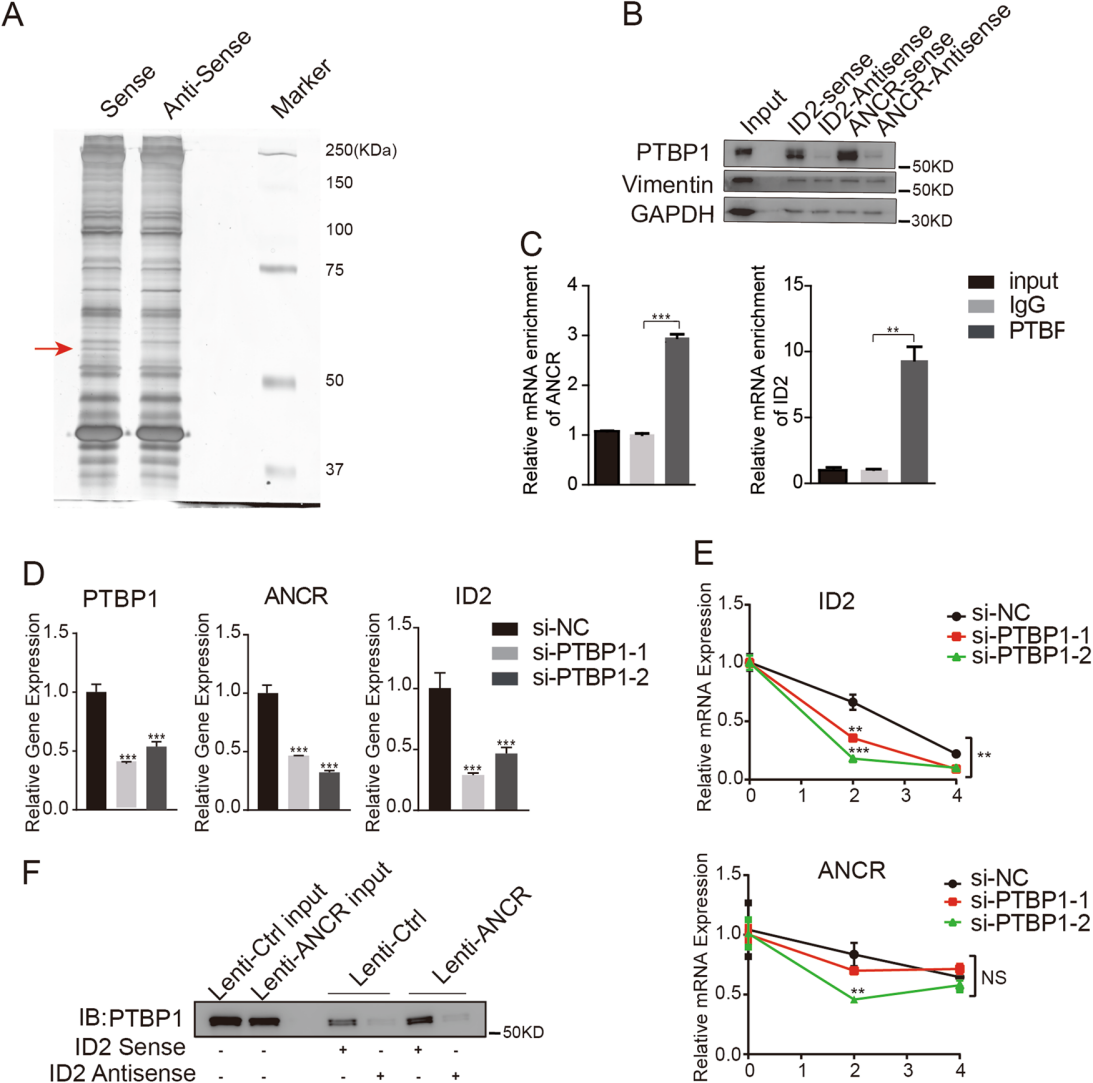
*ANCR* regulates *ID2* mRNA stability by binding to PTBP1. **a** In vitro the RNA pull-down assay. hAMSCs lysates were incubated with biotin-labelled sense or antisense *ANCR* RNAs. After pull down, the proteins were subjected to SDS-PAGE and staining. The band indicated by the arrow was subjected to mass spectrometry. **b** Western blot analysis for PTBP1 or Vimentin following RNA pull down with biotin-labelled sense or antisense *ID2* or *ANCR*. Antisense RNAs incubated with hAMSCs were used as nonspecific controls. **c** The RIP assay for PTBP1 enriched with *ANCR* (left) and *ID2* (right) in hAMSCs. IgG was used as a negative control. All relative abundances were compared to 1% input. **d** PTBP1 was silenced in hAMSCs using two independent siRNAs (si-PTBP1-1 and si-PTBP1-2). The knockdown efficiency and expression of *ANCR* and *ID2* were verified by qRT-PCR compared with si-NC. **e** hAMSCs transfected with si-PTBP1s or si-NC were treated with actinomycin D (5 μg/mL) and RNA was extracted at different time points (0, 2, and 4 h). The levels of *ANCR* and *ID2* were analysed by qRT-PCR and normalized to GAPDH. mRNA at 0 h served as a reference. **f** The RNA pull down assay to determine the interaction between PTBP1 protein and *ID2* mRNA in Lenti-Ctrl or Lenti-ANCR hAMSCs. Data are shown as the means ± S.D. (*n* = 3). *p < 0.05, **p < 0.01, and ***p < 0.001

LncRNAs often regulate target genes through interactions with specific protein partners^37,38^. To explore the underlying mechanism whereby *ANCR* affects *ID2* mRNA stability, we conducted RNA pull-down assays in hAMSCs to identify *ANCR*-interacting proteins. Biotinylated sense and antisense *ANCR* were synthesized and incubated with hAMSC extracts. A discrepant band between sense and antisense lanes (red arrow) was excised, digested, and subjected to mass spectrometry (LC–MS) (Fig. 7a). We chose candidates according to LC–MS scores and validated the interaction by pull-down western blot. The results showed that PTBP1 was identified as a binding partner of *ANCR* (Fig. 7b). PTBP1 (also known as PTB or hnRNP I) is an RNA-binding protein that regulates mRNA stability and pre-mRNA splicing^39^. In contrast, Vimentin, which received the highest score in the LS–MS assay, did not differentially bind to sense and antisense of *ANCR* (Fig. 7b).

To further verify the interaction between PTBP1 and *ANCR*, we performed an RNA immunoprecipitation (RIP) assay in hAMSCs. As shown in Fig. 7c, PTBP1 antibody (anti-PTBP1) significantly precipitated *ANCR* as well as *ID2* compared with an anti-IgG control. Using siRNA silencing to decrease the expression of PTBP1 resulted in decreased levels in both *ANCR* and *ID2* (Fig. 7d). Moreover, knockdown of PTBP1 in hAMSCs could decrease the mRNA stability of *ID2* and *ANCR* when treated with actinomycin D (5 μg/ml) (Fig. 7e). Interestingly, biotin-labelled *ID2* mRNA can also pull down PTBP1 (Fig. 7f). More importantly, overexpression of *ANCR* can significantly increase the binding of PTBP1 and *ID2* mRNA (Fig. 7f). These data collectively revealed that *ANCR* physically binds to PTBP1 and enhanced the interaction between PTBP1 and *ID2* mRNA.

## Discussion

The generation of DE cells with high safety and efficiency is a prerequisite for potential clinical applications. We previously reported a protocol to induce adult adipose-derived MSCs differentiation toward DE and their derived functional cells^8,30^. In this study, we optimized the induction protocol and identified key lncRNAs during the process. We demonstrated that *ANCR* played an important role in the fate conversion of hAMSCs toward DE. *ANCR* could regulate *ID2* mRNA stability by binding to PTBP1 and subsequently suppress the differentiation of hAMSCs into DE in an *ID2*-dependent manner.

The lncRNA *ANCR* was first characterized as a functional suppressor that represses differentiation in epidermal progenitors^23^. *ANCR* has also been reported to modulate osteogenic and adipogenic differentiation in stem cells derived from different tissues^40–42^. In hepatocellular carcinoma, *ANCR/DANCR* was reported to increase stemness features and contribute to tumor progression^43^. Herein, we provided evidence that *ANCR* plays a significant role in suppressing the differentiation of hAMSCs toward DE. We demonstrated that *ANCR* can affect *ID2* mRNA stability during the differentiation of hAMSCs into DE.

Members of the ID family are negative regulators of transcription factors with a basic helix-loop-helix motif^36^. It has been widely observed that ID proteins are abundant in proliferating multipotent cells including stem cell populations, but low or undetectable in differentiated and senescent cells^44,45^. Accumulating studies have revealed that ID proteins play key roles in lineage commitment, cell fate decisions, and oncogenic transformation in cancer^44,46–50^. In MSCs, blocking the degradation of ID proteins through the deubiquitinating enzyme USP1 led to inhibited osteoblastic differentiation and enhanced proliferation^51^. In this study, we found that ID proteins are enriched in hAMSCs and play a negative role in the fate conversion of hAMSCs, which is consistent with the function of *ANCR*. Knockdown of *ID2* by siRNAs promoted DE differentiation from hAMSCs, while ectopic expression of *ID2* exerted opposite effects. Moreover, overexpression of *ID2* could significantly reverse the promoting effects of *ANCR* downregulation on DE differentiation in hAMSCs. The precise mechanism by which *ID2* affects the differentiation of hAMSCs to DE remains to be determined in the future.

PTBP1, which shuttles between the nucleus and cytoplasm, is a multifunctional protein involved in all steps of RNA biogenesis^52^. In the nucleus, PTBP1 forms ribonucleoprotein complexes and regulates alternative splicing, polyadenylation, and mRNA export. In the cytoplasm, PTBP1 participates in localization, translation initiation, and mRNA stability^39^. Here, we demonstrated that lncRNA *ANCR* is mostly distributed in the cytoplasm and could form a complex with PTBP1. We found that PTBP1 knockdown decreased the RNA levels of both *ID2* and *ANCR*. Moreover, *ANCR* overexpression can significantly increase the binding of PTBP1 and *ID2* mRNA. Our findings revealed that PTBP1 is involved in regulating the effects of *ANCR* on *ID2*. Mechanistically, *ANCR* functions as an RNA scaffold to recruit PTBP1 to *ID2*, which modulates *ID2* mRNA stability. Consistently, several lncRNAs have been reported to function in a similar way. LncRNA H19 interacts with PTBP1 to facilitate its association with SREBP1c mRNA and protein, leading to increased stability^53^. LncRNA UCA1 regulates heme biosynthesis and erythrocyte development by recruiting PTBP1 to ALAS2 mRNA^54^.

Interestingly, the expression of *ANCR* is abundant in both ESCs and hAMSCs and remarkably decreased during differentiation into DE. *ANCR* knockdown in hAMSCs enhanced the differentiation efficiency of hAMSCs towards DE. However, *ANCR* depletion in ESCs did not cause similar effects as in hAMSCs. Currently, the precise role of *ANCR* in the regulation of pluripotency and differentiation in ESCs remains unclear. It is suggested that hAMSCs and ESCs might go down different paths and utilize different signaling pathways during the generation of DE. In support of this opinion, we found that BMP and bFGF, which are required for the generation of DE from ESCs^4,32–34^, were dispensable or detrimental to the generation of DE from hAMSCs. BMP signaling promotes DE formation while simultaneously suppressing pluripotency in ESCs/iPSCs^31^. bFGF promotes the epithelial to mesenchymal transition (EMT)^55^, which is a critical step during the acquisition of DE from iPS^56^. We speculated that exit from pluripotency and EMT progress were crucial for DE generation from ESCs/iPSCs but not MSCs. What’s more, the optimal concentration of Activin A for directing MSCs towards DE is relatively low (5 ng/ml), while the optimal concentration for ESCs or iPSCs is high (100 ng/ml)^8^. Most currently available protocols are based on mimicking the DE formation signaling pathway from embryonic development. Our study highlights the importance of initial cell and developing cell source-specific protocols when designing induction schemes.

Collectively, we demonstrate for the first time that the lncRNA *ANCR* can negatively regulate hAMSC differentiation toward DE by binding with PTBP1, enhancing the interaction between PTBP1 and *ID2* mRNA and subsequently increasing *ID2* mRNA stability. LncRNAs are ideal targets for small molecules and nucleic acids because of their specific expression patterns and unique sequences and secondary structure^57^. Further identification of functional lncRNAs as well as cell type-specific signaling in the generation of DE cells will help to develop new strategies to enhance the efficiency of cell fate conversion or differentiation.

## Materials and methods

### Isolation, culture, and differentiation of hAMSCs

hAMSCs were isolated from human adipose tissues obtained from donors undergoing liposuction according to our previous studies^8,30^. hAMSCs at passage 3 were used in our experiments. All experiments and procedures were approved by the Ethics Committee at the Chinese Academy of Medical Sciences and Peking Union Medical College.

For DE differentiation, as described previously^8^ and in Supplementary Fig. 1a, hAMSCs at passage 3 were seeded in six-well plates in regular culture medium. The next day, the cells were changed to differentiation basic medium DMEM (Gibco, Grand Island, NY) supplemented with 0.5% FBS (Gibco, Grand Island, NY), 5 ng/ml Activin A (Peprotech, USA), and 50 ng/ml Wnt3a (Peprotech, USA)(AW) or 0.3 µM Chir99021(AC) on the first days, with Wnt3a or Chir99021 being withdrawn on the following 4 days. For the comparison with ESC protocol published by Hannan et al.^4^ (H), BMP4 (10 ng/ml) and bFGF (10 ng/ml) were added to the differentiation system on the first two days.

### Culture and differentiation of ESCs

The embryonic stem cell line H9 was kindly provided by Dr Du Mingxia (Peking Union Medical College, Beijing, China). H9 cells were cultured on Matrigel (BD Biosciences, San Jose, CA) with a mTeSR1 Complete Kit (Stem Cell Technology, Canada) and passaged every 3–5 days using 0.05 mM EDTA (Cell APY, Beijing, China) according to the manufacturer’s instruction.

For DE differentiation, as described in Fig. S2a^12^, H9 cells were passaged with Accutase (Life Technologies, USA) and seeded on Matrigel (BD Biosciences, USA)-coated six-well plates at a density of 1–2 × 10^5^ cells per well. The next day, the medium was changed to CDM-PVA supplemented with 100 ng/ml Activin A, 100 ng/ml bFGF (Peprotech, USA), 10 ng/ml BMP4 (Peprotech, USA), and 0.3 µM Chir99021 (Selleck, USA) for 1 day. On day 2, Chir99021 was withdrawn. From day 3 to 5, the medium was changed to RPMI1640 (Life Technologies, USA) supplemented with 100 ng/ml Activin A (Peprotech, USA) and 50 ng/ml bFGF (Peprotech, USA).

### Microarray analysis

To identify DE-related lncRNAs, hAMSCs were exposed to DE induction medium as described above, and total RNA was isolated using the Trizol reagent (Invitrogen, USA) on days 0, 3, and 5. hAMSCs from three donors were analysed in this study. Sample processing and hybridization were conducted by Cnkingbio Biotechnology (China) with Affymetrix mRNA + lncRNA microarray chips. Briefly, a fold change ≥ 2 (expression value ≥3 and *p* value < 0.05, day 3 and 5 versus day 0) were chosen as the cutoff criteria for differentially expressed genes. Overlapping DEGs in all three donors were used in GO enrichment analyses.

For *ANCR*-affected genes, RNA was extracted from hAMSCs transfected with si-NC (negative control), si-ANCR1, and si-ANCR2 for 48 h. hAMSCs from two donors were analysed in this study. A fold change ≥2 over the control, an expression value ≥4 and a *p* value < 0.05 were chosen as the cutoff criteria for differentially expressed genes.

### siRNA and lentivirus infection

siRNAs used to knockdown target lncRNAs or mRNAs were designed using online tools (BLOCK-iT™ RNAi Designer) and synthesized by the RIBOBIO company (Suzhou, China). For transfection of the siRNAs, Lipofectamine 2000 (Life Technology, USA) was used according to the manufacturer’s recommendations.

For overexpression, full-length *ANCR* and *ID2* CDS were inserted into the pEZ-LV225 lentivirus expression vector and packaged by GeneCopoeia^TM^. hAMSCs were infected with viral precipitates at an MOI of 10 and stable cell lines were established by puromycin treatment.

### RNA extraction and qRT-PCR analysis

Total RNA was extracted using the Trizol Regent (Invitrogen, USA), and 2 μg of RNA were reverse transcribed with oligo (dT) primer and M-MLV Reverse Transcriptase (Takara, Japan). qRT-PCR was performed on a QuantStudio™ Design & Analysis system (ABI, USA) with SYBR-Green Mastermix (YEASEN, China). The relative RNA levels were normalized to GAPDH using the 2^−ΔΔCt^ method. The primer sequences are listed in Supplementary Table 1.

### Western blot

Protein was extracted using RIPA buffer with PMSF (1:100, Beyotime, China) and quantified with a BCA Protein Assay kit (Beyotime, China). Proteins in lysates were separated by 10% SDS-PAGE and transferred to polyvinylidene difluoride membranes (0.22μm, Millipore, Danvers, MA, USA). The membranes were blocked with 5% milk for 1h at room temperature, incubated with primary antibody overnight at 4 °C, and then incubated with horseradish peroxidase-conjugated secondary antibodies (1:3000, YEASEN, China) at room temperature for 1h. The proteins were detected using an ECL reagent (Millipore, USA).

### Immunofluorescent staining

Cells were fixed with 4% paraformaldehyde, permeabilized in 0.3% Tween-100, blocked in PBS + 0.5% Tween-100 + 5% BSA, and then incubated with primary antibody overnight at 4 °C. After washing three times with PBS, the sample was incubated with the corresponding secondary antibody at room temperature for 1 h, washed with PBS, and then incubated with Hoechst for 5min to dye the nuclei. The antibodies used in this study are summarized as follows.

The OCT4 and FOXA2 antibodies were obtained from Abcam (Cambridge, MA, USA); SOX17 was bought from Cell Signaling Technology (CST, USA); Hoechst was purchased from Solarbio (Beijing, China); and Alexa Fluor 488 goat-anti-rabbit secondary antibody and Alexa Fluor 594 goat-anti-mouse secondary antibody were bought from Thermo Fisher Scientific (Waltham, MA, USA).

### The flow cytometry assay

The differentiated cells were analysed for cell antigen expression by flow cytometry using an Accuri C6 (BD Biosciences, San Jose, CA). In total, 1 × 10^5^ cells were fixed in 4% paraformaldehyde, permeabilized with PBS + 0.2% Triton X-100 for 10 min, and then incubated at 4 °C for 1 h with the following primary antibodies: SOX17, FOXA2, or isotype antibodies, which served as negative controls. Then, the cells were incubated with the following antibodies: goat-anti-rabbit-488 or goat-anti-mouse-594 at room temperature for 30 min.

### mRNA stability analysis

hAMSCs were firstly transfected with si-NC and si- ANCR or si-PTBP1 for 48 h. Then, the hAMSCs were treated with 5 μg/ml actinomycin D (MedChemExpress, NJ, USA). At different time points (0, 2, and 4 h), total RNA was extracted using the Trizol regent and *ANCR* or *ID2* mRNA was analysed by qRT-PCR and normalized to *GAPDH*. mRNA at 0 h served as a reference.

### Subcellular fractionation

The separation of the nuclear and cytosolic fractions was performed using the NE-PER Nuclear and Cytoplasmic Extraction Reagents (Thermo Fisher Scientific, USA) according to the manufacturer’s instructions. RNA was extracted, and qRT-PCR was performed to assess the relative proportion in the nuclear and cytoplasmic fractions.

### RNA-pull down and mass spectrometry

Full-length *ANCR* and *ID2* were synthesized and subcloned into the pCI-neo vector. Biotin-labelled RNAs were transcribed in vitro with the Biotin RNA Labeling Mix (Roche, Basel, Switzerland) and T7 RNA Pol II (NEB, USA). Whole-cell extracts prepared from 1 × 10^7^ hAMSC cells (empty vector control or *ANCR* overexpression) in 1 ml of RIP buffer (150 mM KCl, 25 mM Tris (pH 7.4), 5 mM EDTA, 0.5 mM DTT, and 0.5% NP-40) containing RNase and protease inhibitors was mixed with 3 µg of biotinylated RNA and incubated at RT for 2 h, followed by the addition of 50 µl washed Streptavidin Dynabeads to incubate for another 1 h. After magnetic separation, beads were washed four times with ice-cold buffer and resuspended in 1× SDS sample buffer. The precipitated components were separated using SDS-PAGE, followed by silver staining. Differential bands were cut for mass spectrometry (LTQ Orbitrap XL).

### The RIP assay

The RIP assay was performed with the EZ-Magna RIP Kit (Millipore, USA) according to the manufacturer’s instructions. Anti-PTBP1 antibody was purchased from Proteintech (Wu Han, China). The coprecipitated RNAs associated with PTBP1 were extracted with the Trizol reagent, and *ANCR* and *ID2* enrichment was examined using RT–qPCR. Enrichment associated with normal rabbit IgG served as controls.

### Statistical analysis

GraphPad Prism7 (GraphPad Prism, San Diego, CA) software was used for all statistical analysis and expressed as mean ± standard. Student’s *t*-test was used for statistical comparison between two groups. One-way ANOVA was used for comparison between multiple groups. Differences were considered statistically significant at *P < 0.05, **P < 0.01, and ***P < 0.001.

## Supplementary information

Detailed attribution of authorship

Supplementary Table 1

Supplementary Figure legends

Supplementary Figure 1

Supplementary Figure 2

Supplementary Figure 3
