# Supplementary material for: Long noncoding RNA ANCR inhibits the differentiation of mesenchymal stem cells toward definitive endoderm by facilitating the association of PTBP1 with ID2
Source: Cell Death Dis. 2019 Jun 24;10(7):492. doi: 10.1038/s41419-019-1738-3 (PMC6591386; doi:10.1038/s41419-019-1738-3)
Supplement: Supplementary file 1 — Detailed attribution of authorship [file 41419_2019_1738_MOESM1_ESM.docx]

**Author contributions**

JL and YY designed, performed experiments, analyzed the data, prepared the figures and drafted the paper; JF, HY and LY performed some experiments and analyzed the data; HL organized, designed, and revised the paper, and RC initiated the study.
