# Supplementary material for: Long noncoding RNA ANCR inhibits the differentiation of mesenchymal stem cells toward definitive endoderm by facilitating the association of PTBP1 with ID2
Source: Cell Death Dis. 2019 Jun 24;10(7):492. doi: 10.1038/s41419-019-1738-3 (PMC6591386; doi:10.1038/s41419-019-1738-3)
Supplement: Supplementary file 2 — Supplementary Table 1 [file 41419_2019_1738_MOESM2_ESM.docx]

**Supplementary Table 1. Sequence of primers and siRNAs used in this study.**

| **Gene** | **Primer sequence** (5’-3’) |
| --- | --- |
| *OCT4* | F: GCTCGAGAAGGATGTGGTCC |
|  | R: CGTTGTGCATAGTCGCTGCT |
| *NANOG* | F: GCAGAAGGCCTCAGCACCTA |
|  | R: AGGTTCCCAGTCGGGTTCA |
| *SOX17* | F: GCATGACTCCGGTGTGAATCT |
|  | R: TCACACGTCAGGATAGTTGCAGT |
| *FOXA2* | F: CTGAGCGAGATCTACCAGTGGA |
|  | R: CAGTCGTTGAAGGAGAGCGAGT |
| *CXCR4* | F: ACTACACCGAGGAAATGGGCT |
|  | R: CCCACAATGCCAGTTAAGAAGA |
| *GSC* | F: AACGCGGAGAAGTGGAACAAG |
|  | R: GTCCAAATCGCTTTTACCTTCCT |
| *EOMES* | F: ATCATTACGAAACAGGGCAGGC |
|  | R: CGGGGTTGGTATTTGTGTAAGG |
| *PAX6* | F: GCCCAGCTTCACCATGGCAAATAA |
|  | R: ATCATAACTCCGCCCATTCACCGA |
| *KDR* | F:AGTGATCGGAAATGACACTGGA |
|  | R: GCACAAAGTGACACGTTGAGAT |
| *ANCR* | F: CCTCTTTGTCAGCTGGAGTT |
|  | R: ATGGCTTGTGCCTGTAGTT |
| *ID2* | F: AATAGTGGGATGCGAGTCCAG |
|  | R: GCTATACAACATGAACGACTGCT |
| *PTBP1* | F: AGCGCGTGAAGATCCTGTTC |
|  | R: CAGGGGTGAGTTGCCGTAG |
| *GAPDH* | F: GGTCACCAGGGCTGCTTTTA |
|  | R: GGATCTCGCTCCTGGAAGATG |
| si-NC | r(UUCUCCGAACGUGUCACGU)dTdT |
| si-*ANCR*-1 | r(GCCAACTATCCCTTCAGTT)dTdT |
| si-*ANCR*-2 | r(GCCGGTCATGAGATTATAT)dTdT |
| si-*PTBP1*-1 | r(GCGTGAAGATCCTGTTCAA) dTdT |
| si-*PTBP1*-2 | r(GCCTCAACGTCAAGTACAA) dTdT |
| si-*ID2*-2 | r(CGATGAGCCTGCTATACAA) dTdT |
| si-*ID2*-3 | r(GGACTCGCATCCCACTATT) dTdT |
