# Supplementary material for: Long noncoding RNA ANCR inhibits the differentiation of mesenchymal stem cells toward definitive endoderm by facilitating the association of PTBP1 with ID2
Source: Cell Death Dis. 2019 Jun 24;10(7):492. doi: 10.1038/s41419-019-1738-3 (PMC6591386; doi:10.1038/s41419-019-1738-3)
Supplement: Supplementary file 3 — Supplementary Figure legends [file 41419_2019_1738_MOESM3_ESM.docx]

**Supplementary** **Figure1. Protocol to differentiate hAMSCs into DE.**

1. Schematic overview of the differentiation protocol to generate DE from hAMSCs. Exposure of hAMSCs in basic medium (BM) supplemented with 5 ng/ml Activin A and 50 ng/ml Wnt3a (AW) or Chir99021(AC) from day 0 to day 1, and then in BM supplemented with 5 ng/ml Activin A from days 2 to 5 resulted in the formation of DE cells (FOXA2^+^ and SOX17^+^).
2. qRT-PCR analysis of the DE markers (*FOXA2*, *SOX17* and *CXCR4*) in hAMSCs after DE induction using the AW or AC protocol.

**Supplementary Figure2. Validation of the differentiation of ESCs into DE.**

(A) Schematic overview of the differentiation protocol to generate DE from ESCs ^4^ (protocol H for short).

(B**)** qRT-PCR analysis detected DE marker genes (*SOX17* and *FOXA2*)，mesendoderm marker genes (*EOMES* and *Brachyury*) and stem cell marker genes (*OCT4* and *NANOG*) in ESCs at the indicated time points after DE induction.

(C) Immunofluorescence staining of OCT4, SOX17 and FOXA2 in ESCs and DE. Hoechst (blue), OCT4 (green), SOX17 (green) and FOXA2 (red). Scale bar = 100μm.

**Supplementary Figure3.** **BMP and bFGF were dispensable in the conversion of hAMSCs to DE.**

(A) Comparison of the schematic overview of DE differentiation between ESCs and hAMSCs.

(B) qRT-PCR analysis compared the DE markers (*SOX17* and *FOXA2*) from protocols H, AC and AC+BMP4.

(C) qRT-PCR analysis compared the DE markers (*SOX17* and *FOXA2*) from protocols H, AC and AC+bFGF.
