# Supplementary figures and images for: Long noncoding RNA ANCR inhibits the differentiation of mesenchymal stem cells toward definitive endoderm by facilitating the association of PTBP1 with ID2
Source: Cell Death Dis. 2019 Jun 24;10(7):492. doi: 10.1038/s41419-019-1738-3 (PMC6591386; doi:10.1038/s41419-019-1738-3)

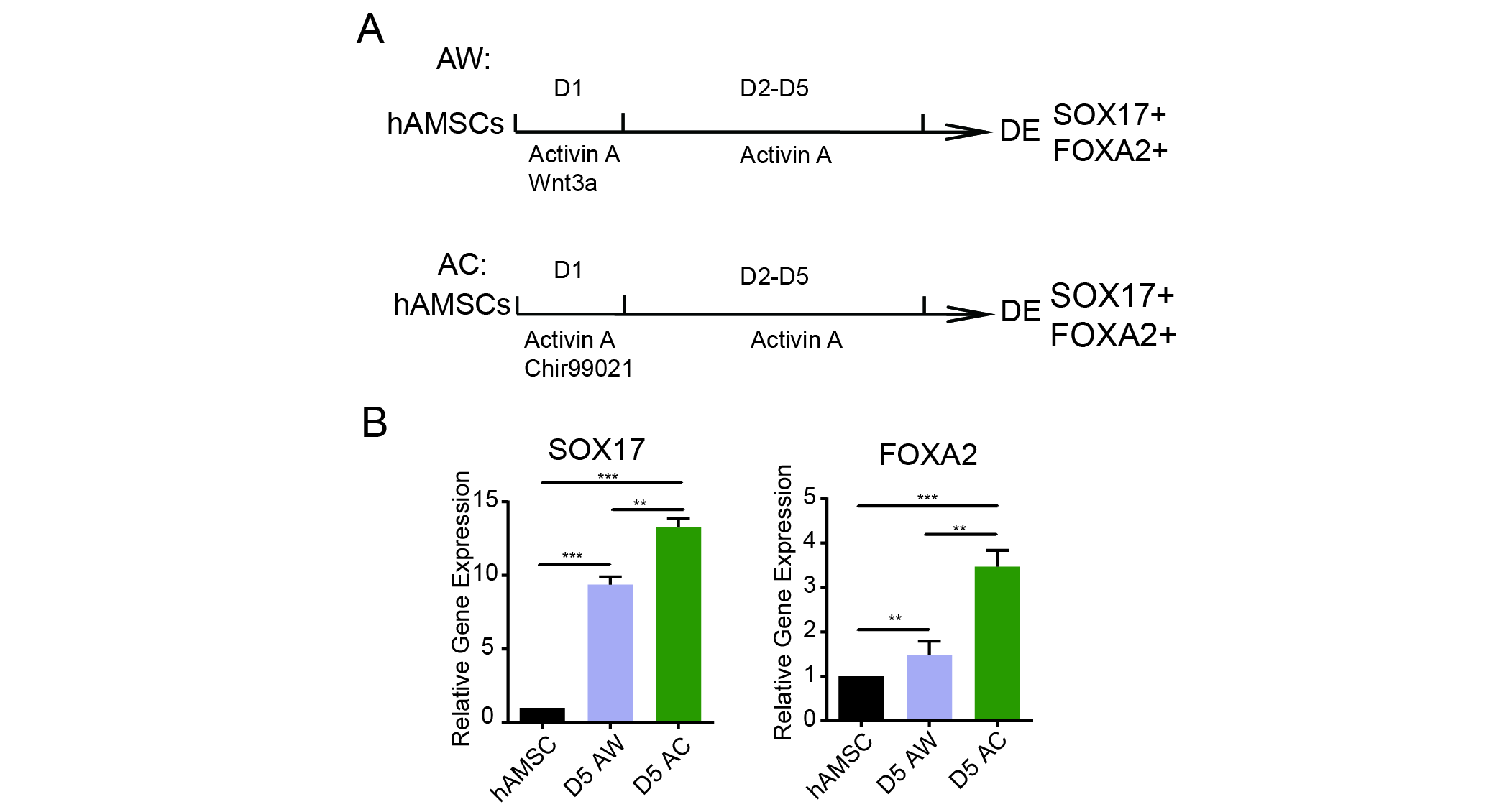

Supplement: Supplementary file 4 — Supplementary Figure 1 [file 41419_2019_1738_MOESM4_ESM.tif]

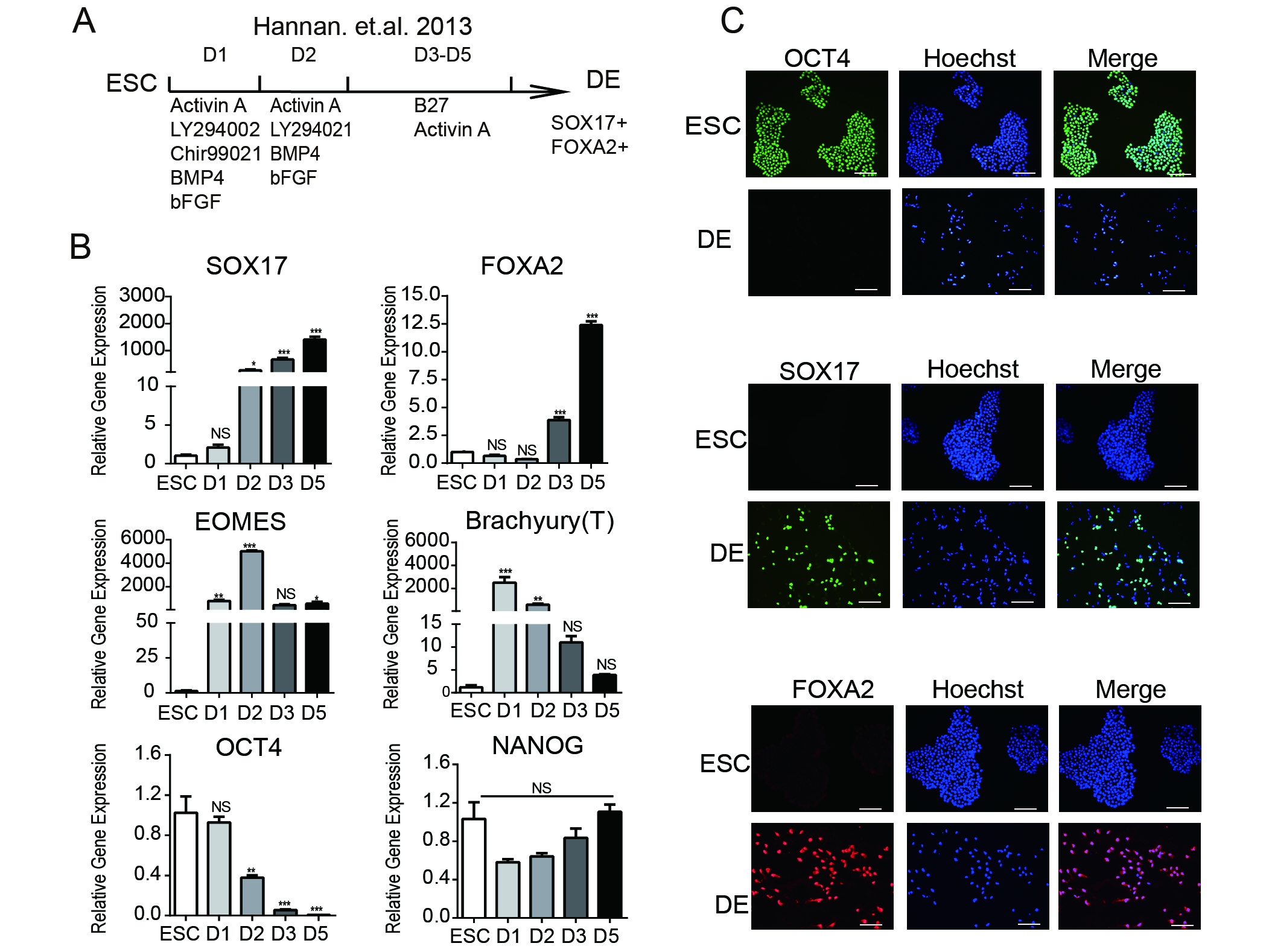

Supplement: Supplementary file 5 — Supplementary Figure 2 [file 41419_2019_1738_MOESM5_ESM.tif]

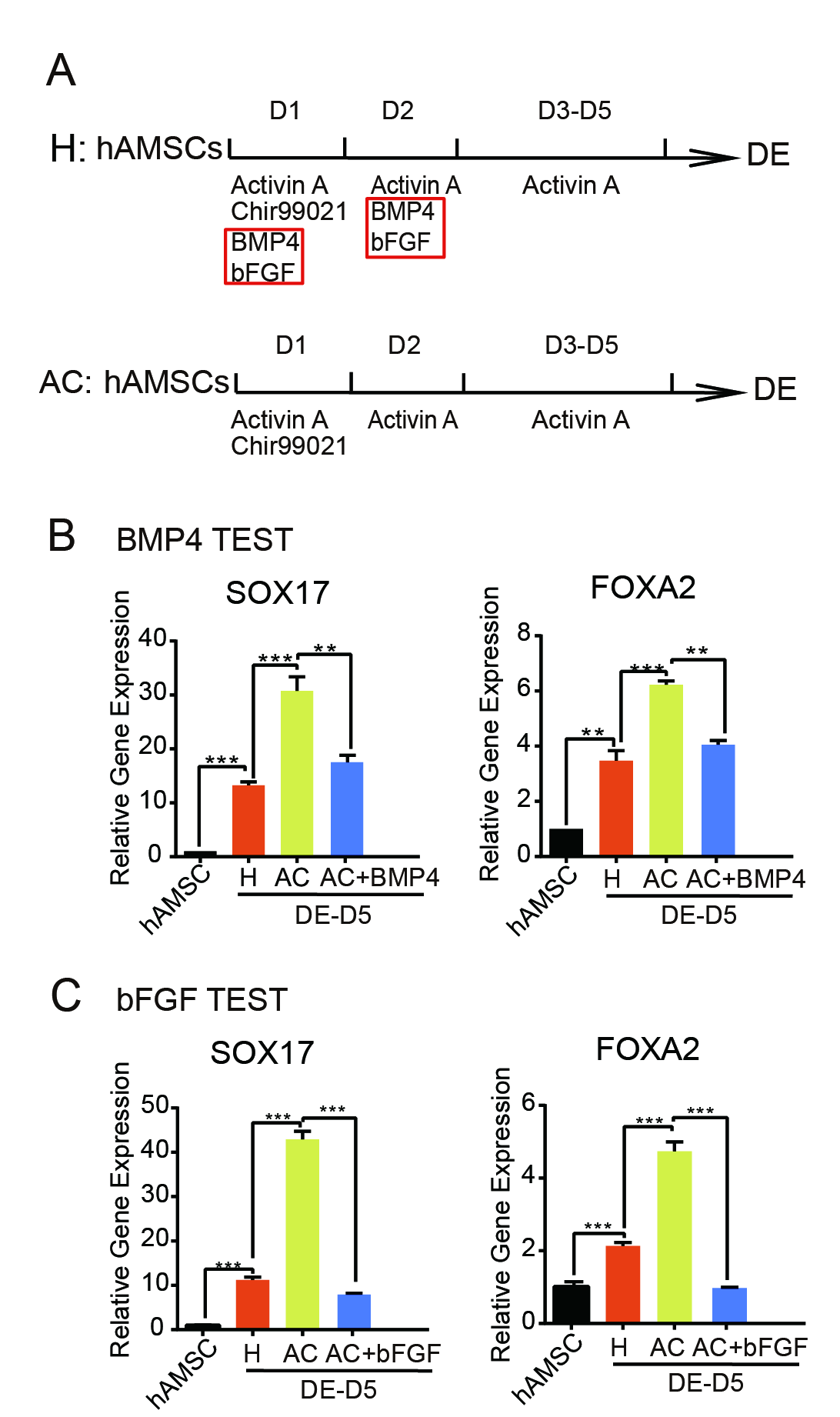

Supplement: Supplementary file 6 — Supplementary Figure 3 [file 41419_2019_1738_MOESM6_ESM.tif]
